# Influence of Oral Intaking Habit on Tongue Coating Microbiota in Patients with Esophageal Precancerous Lesions

**DOI:** 10.7150/jca.67068

**Published:** 2022-01-16

**Authors:** Pan Xiao, Zhaolai Hua, Xiaoyu Kang, Bin Lu, Meifeng Li, Juan Wu, Wei Dong, Junfeng Zhang, Chun Cheng

**Affiliations:** 1 School of Medicine & Holistic Integrative Medicine, Nanjing University of Chinese Medicine, Nanjing 210023, Jiangsu, China; 2 Yangzhong Cancer Institute, Yangzhong People's Hospital, Jiangsu Yangzhong 212200, China; 3 Department of Oncology, Yangzhong People's Hospital, Yangzhong 212200, Jiangsu, China

**Keywords:** esophageal precancerous lesions, tongue coating, microbiota, oral intaking habit, bile acid

## Abstract

**Background:** Esophageal cancer (EC) is a common digestive tract tumor in China, and oral intaking habit has a great influence on the development of EC. The present study explored the correlation between oral intaking habit and tongue coating (TC) microbiota in patients with esophageal precancerous lesions (EPL) to provide a reasonable interpretation of the influence of oral intaking habit on microbial alterations in the EPL.

**Methods:** A case-control study was designed with 123 EPL patients and 176 volunteers with mild esophagitis, and they were well matched using sex, age, and body mass index. The TC microbiota was profiled using high-throughput sequencing of the V3-V4 region of the 16S rRNA gene, and the serum levels of total bile acid (TBA) and interleukin-17α (IL-17α) were measured using enzyme-linked immunosorbent assay. Alpha diversity, community structure, and linear discriminant analysis were conducted, and Spearman correlation analysis was used to build the symbiotic network.

**Results:** No significant differences were observed in the diversity and richness of the TC microbiota between the cases and controls (*P* > 0.05). TC *Peptostreptococcus* and *Capnocytophaga* were enriched in EPL patients. Stratified analysis showed that TC microbial composition was affected by both EPL and oral intaking habit; for example, *Atopobium* and *Actinomyces* were positively related to oral intaking habit scores in both the cases and controls, while* Simonsiella* was negatively correlated with oral intaking habit status in cases but positively correlated with oral intaking habit status in controls. Although serum TBA and IL-17α were not associated with EPL (*P* > 0.05), the daily-drinking cases had a higher level of serum TBA than the nondrinking cases (*P* < 0.05), and *Helicobacter pylori* (Hp) negative controls had a higher level of serum TBA than the Hp-positive controls (*P* < 0.05). The symbiotic networks were comprised of 71 significant correlations in the controls and 52 significant correlations in the cases.

**Conclusions:** The development of EPL changed the TC microbiota and decreased the symbiotic complexity of the TC bacteria, which were also influenced by the cancer-related oral intaking habit. Bile acid may be a key factor mediating changes in TC microbiota.

## Introduction

Esophageal cancer (EC), which originates from the esophageal mucosal epithelium, is the 7th most common malignancy and the 6th leading cause of cancer mortality in both sexes combined worldwide, with approximately 572,000 new cases and 509,000 deaths in 2018. Among them, the incidence (13.9/100,000) and mortality (12.7/100,000) of EC in China was ranked fifth and fourth, respectively, among malignant tumors, with 53.7% and 55.7% of the total worldwide 1, 2. According to a recent report 3, the incidence (11.28/100,000) and mortality (8.36/100,000) of EC ranked sixth and fourth among malignant tumors in China in 2015. EC is a characteristic high incidence malignant tumor in China, and Jiangsu Province is a typical high incidence area with crude incidence of (36.4/100,000) and crude mortality of (28.4/100,000) in 2015. Despite a downward trend in the standardized rate of EC from 2006-2015, the overall crude morbidity and crude mortality rates remained unchanged, and the crude morbidity and gross mortality rates in the rural population continue to mount with the accelerating aging process of the social population, making the challenge of managing EC difficult 4. Improving the early diagnosis, treatment, and screening of patients with esophageal precancerous lesions (EPL) could significantly prolong the survival rate and reduce the mortality of patients with EC 5.

EPL develops gradually from precancerous lesions, and it takes years for precursor lesions to develop cancer. According to the pathological development process, EPL experienced basal cell hyperplasia, mild dysplasia, moderate dysplasia, and severe dysplasia/carcinoma in situ 6. Esophageal squamous cell dysplasia can develop into esophageal squamous cell carcinoma (ESCC), which is the most common pathological type of EC in China 7, while Barrett's esophagus-associated dysplasia can develop into esophageal adenocarcinoma (EAC), which is relatively rare in China 8. The occurrence and development of ESCC is a long-term, slow process involving many factors and stages. Many risk factors of EC, such as aging, male sex, family history, smoking, drinking, fried, and salty food, have been identified via epidemiological investigations 9-11. In the real world, the burden of EC in urban areas is lower than that in rural areas, which may be related to education level, living environment, smoking, and dietary patterns 12. Our series of studies found that oral intaking habit was the main factor influencing the sex difference in the risk of gastric cancer in East China (such as Jiangsu Province) 13, tongue coating (TC) bacteria, but not fungi, could be used as potential noninvasive biomarkers to identify patients with gastric cancer 14, 15.

TC is a thin white and moist coating located on the tongue dorsum and composed mainly of tree-like keratinized nipples with filamentous papillae, shed epithelial cells, saliva, bacteria, food residue, and exudative white blood cells. We reviewed the application value of TC microbiota and proposed that TC microbiota is the most important distribution center in oral cavity microbiota and plays a key role in the development of many digestive system tumors, including gastric carcinoma, pancreatic cancer, liver cancer, and colorectal cancer 16. Thus, this study aimed to explore the association among EPL risk, TC microbiota, and oral intaking habit to propose a new approach to managing EPL using the noninvasive TC microbiota.

## Materials and methods

### Participants

From April 2019 to January 2020, a uniform questionnaire was used to collect clinical data for upper gastrointestinal cancer screening among residents aged 40-70 in Yangzhong City. Based on the consensus criteria for gastroscopic pathological diagnosis and precancerous lesions 17, 123 EPL patients were enrolled. A total of 176 well-matched volunteers with mild esophagitis were recruited as controls, and informed consent was obtained from all participants. The exclusion criteria for the cases were as follows: 1) history of malignant tumors; 2) complications of severe infectious diseases or oral mucosa lesions within 5 years; 3) Barrett's esophagus; 4) use of antibiotics or proton pump inhibitors within 4 weeks; and 5) unwillingness to cooperate. The exclusion criteria for the controls were as follows: 1) history of malignant tumors; 2) history of serious digestive system diseases (such as chronic atrophic gastritis, gastric ulcer, ulcerative colitis, and hepatitis) (self-reported); 3) obvious symptoms of digestive tract discomfort, such as stomach pain, abdominal pain, and diarrhea (self-reported); 4) use of antibiotics or proton pump inhibitors within 4 weeks; and 5) unwillingness to cooperate. The study protocol was approved by the Clinical Ethics Committee of the People's Hospital of Yangzhong City (No. PHYC2018039).

### Sample collection

All participants were asked to fast overnight (≥ 8 h) and to rinse their mouths with sterile saline buffer 2-3 times. Each TC sample was collected from the middle section of the tongue dorsum using a sterile one-off toothbrush and placed in a test tube with sterile saline. The tubes were centrifuged at 3000 rpm for 5 min, and the precipitates were collected. At the same time, 5 mL peripheral blood was collected, and the serum was centrifuged at 3000 rpm for 10 min. The samples were immediately stored at -80 °C.

### Sample sequencing

The TC microbiota was detected by Shanghai Lingen Biotechnology Co., Ltd. The detection method was based on the previous literature 18 and is briefly described as follows: The total DNA of the TC precipitation was extracted using the V3-V4 region of the 16S rRNA gene universal primer (341F-5´-CCTAYGGGRBGCASCAG-3´, 806R-5´-GGACTACNNGGGTATCTAAT-3´). A polymerase chain reaction amplification was performed, a Miseq PE library was prepared, and high-throughput sequencing was performed. The valid sequence was obtained and the microbiota operational taxonomic units (OTUs) were established in the UPARSE35 software (version 7.1 http://drive5.com/uparse/) according to a 97% similarity threshold. The Visual Genomics software (Release 1, Shanghai Infinity Biotechnology Co., Ltd.) was used to analyze the biological information of the microbiota.

### Laboratory testing

A rapid urease test 19 was used to detect the infection status of *Helicobacter pylori* (Hp) in the stomach during a gastroscopy (Guangzhou Beisiqi Reagent Co., Ltd.). Total bile acid (TBA) (TBA ELISA Kit, MM-50350H1) and interleukin-17α (IL-17α) (Human IL-17α ELISA Kit, ZC-32330) were detected using enzyme-linked immunosorbent assay (ELISA). All operations were carried out in strict accordance with the kit instructions and the specific ELISA methods referred to in previous studies 20.

### Statistical analysis

The statistical software (SPSS 26.0) was used to analyze and process the data. The data of normal distribution are presented as the mean ± standard deviation. The comparison between two independent samples was analyzed using a *t*-test, and the differences among groups were analyzed using the Mann-Whitney *U* test. The grade data were analyzed using the chi-square test. Correlations between species were analyzed using Spearman's correlation coefficient. Cytoscape (version 3.7.0) was used to visualize bacterial co-occurrence. The correlation heatmap was drawn using R software packages, and a point map was drawn using GraphPad Prism 8 software. All tests for significance were two-sided, and *P-*values < 0.05, were considered significant.

## Results

### Participant characteristics and overall sequencing data

In this study, the population consisted of 123 EPL patients and 176 controls. There were no significant differences in age, body mass index, sex, salty food, fried food, regular meals, drinking tea, and Hp infection between the two groups (*P* > 0.05). It is worth noting that the proportion of daily drinking in cases was significantly higher than that in controls (*P* < 0.05), while the proportion of daily smoking in cases was significantly lower than that in controls (*P* < 0.05) **(Table 1)**.

All the 299 TC samples were sequenced. For the bacterial 16S rRNA gene V3-V4 region, the total number of sequences was 13,858,569, with an average of 46,350 reads per sample, the average length of the reads was approximately 46,350 bp, and 5,190 OTUs were detected.

### Diversity analysis

To assess the diversity of microbiota in the cases and controls of TCs, ACE, Chao, Shannon, and Simpson indices were used to describe the alpha diversity. The ACE and Chao indices reflect the richness of OTUs, whereas the Shannon and Simpson indices were used to assess the diversity of OTUs. However, there were no significant differences in microbial richness and diversity between the cases and controls (*P* > 0.05) **(Table 2)**. These results indicate that the variation in TC is irrelevant to the richness and diversity of the microbiome community in EPL patients.

### Community structure of TC microbiota

**Figure 1:**
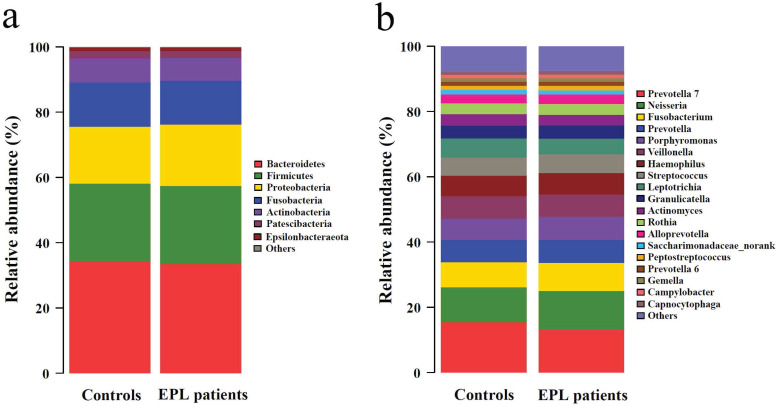
Community structure of TC microbiota at phylum-level (a) and genus-level (b).

The 5,190 bacterial OTUs from the TC samples were clustered into 11 phyla, 19 classes, 44 orders, 86 families, and 191 genera. The dominant phyla (relative abundance > 1%) included Bacteroidetes, Firmicutes, Proteobacteria, Fusobacteria, Actinobacteria, Patescibacteria, and Epsilonbacteraeota **(Figure 1a, Supplementary data for Figure 1a)**. The dominant genera (average relative abundance > 1%) consisted of *Prevotella 7*, *Neisseria*, *Fusobacterium*, *Prevotella*, *Porphyromonas*, *Veillonella*, *Haemophilus*, *Streptococcus*, *Leptotrichia*, *Granulicatella*, *Actinomyces*, *Rothia*, *Alloprevotella*, *Saccharimonadaceae_norank*, *Peptostreptococcus*, *Prevotella 6*, *Gemella*, *Campylobacter*, and *Capnocytophaga* **(Figure 1b, Supplementary data for Figure 1b)**.

The Mann-Whitney *U* test showed that no significantly different phyla were observed between the cases and controls (*P* > 0.05), and nine significantly different genera (*P* < 0.05) are shown in **Table S1**. Compared with controls, the relative abundances of *[Eubacterium] yurii group*,* Capnocytophaga*,* Comamonas*,* Defluviitaleaceae UCG-011*, *Odoribacter*, and* Peptostreptococcus* in the TC of EPL patients increased significantly, while the relative abundances of *Atopobium*, *Hydrobacter*, and *Taonella* decreased significantly.

### Microbial biomarkers of EPL patients

**Figure 2:**
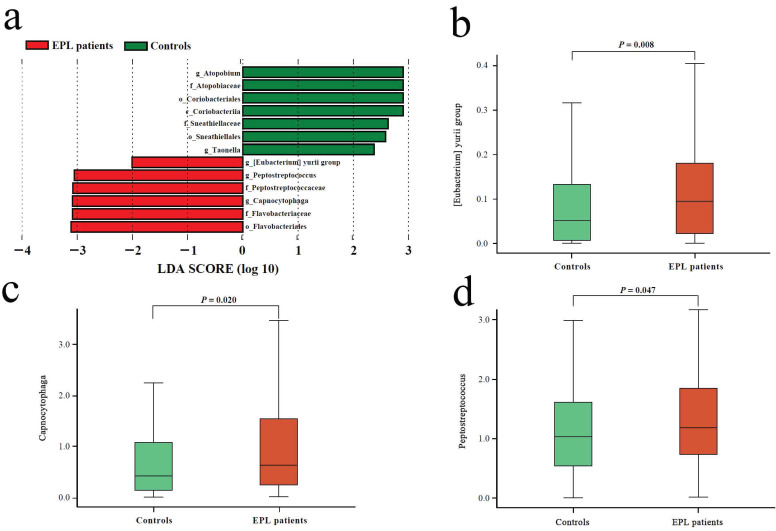
** Microbial biomarkers of EPL patients.** (**a**) LEfSe analysis between the controls and cases. The different analysis of three genera including *[Eubacterium] yurii group* (**b**), *Peptostreptococcus* (**c**), and *Capnocytophaga* (**d**).

Linear discriminant analysis (LDA) was performed at the phylum to genus level to explore the TC microflora associated with EPL risk. The results showed that six bacterial taxa were enriched in the cases and seven bacterial taxa were enriched in the controls **(Figure 2a)**. Among them, the relative abundances of *[Eubacterium] yurii group*, *Peptostreptococcus*, and* Capnocytophaga* were significantly higher in cases than in controls (*P* < 0.05) **(Figures 2b, 2c, and 2d)**, which may be potential microbial biomarkers for EPL in the TC sample.

In addition, the study found that the status of Hp infection also affected the TC microbiota. In the control group, 11 bacterial taxa (including Firmicutes, Bacilli, Lactobacillales, etc.) were enriched in the Hp-negative population; in cases, 1 bacterial taxon (*Prevotella 2*) were enriched in the Hp-positive population and four bacterial taxa (Actinobacteria, Lachnoanaerobaculum, Labraceae, and *Labrys*) were enriched in the Hp-negative population **(Figure S1)**.

### Symbiotic networks of TC microbiota

**Figure 3:**
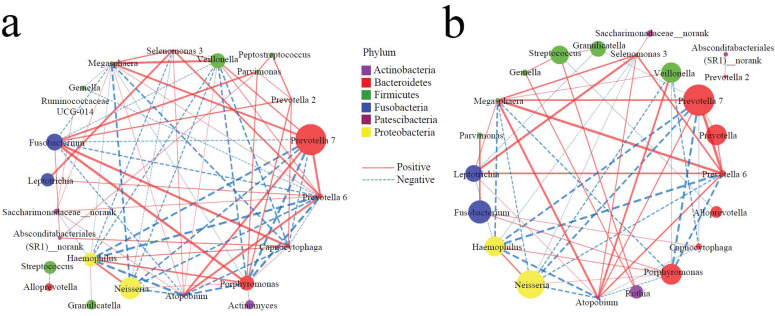
** Symbiotic networks of TC microbiota**. The dominant bacteria with a relative abundance of more than 1% in the total population were selected, and the Spearman correlation analysis was performed in controls (**a**) and cases (**b**) to reveal the genera of the dominant bacteria with significant association (*P* < 0.05). The node size indicates relative abundance and the node color indicates the phylum; the solid line indicates a significant positive correlation, the dotted line indicates a significant negative correlation, and the thickness of the line indicates the correlation coefficient.

It is well known that in the healthy state, bacteria have a complex symbiotic relationship and maintain a normal micro-ecological balance; in the disease state, the symbiotic relationship of bacteria changes significantly, resulting in dysbacteriosis. Therefore, the functional relationship of the TC microbiota may provide a new entry point for understanding the pathophysiological mechanisms underlying the occurrence of diseases. Based on the top 30 genera, Spearman correlation analysis was conducted to construct symbiotic networks. In the controls, there were 39 positive and 32 negative correlations **(Figure 3a, Supplementary data for Figure 3a),** and there were 29 positive and 23 negative correlations in the cases **(Figure 3b, Supplementary data for Figure 3b)**. There was a significant positive correlation between *Peptostreptococcus* and *Parvimonas* /* Prevotella* 2 in the controls, but a weak correlation in the cases. On the other hand, several genera (*Fusobacterium*, *Porphyromonas*, *Haemophilus*, *Absconditabacteriales (SR1)_norank*, *Prevotella 2*) were positively correlated to *Capnocytophaga* in the controls (r > 0.5), while there were several nodes involved in negative networks in the cases, such as *Prevotella 7*,* Prevotella 6*,* Megasphaera*, and* Atopobium*. These results suggest that the occurrence of EPL weakened the symbiotic network and reduced the symbiotic complexity of the TC microbiota.

### Influence of oral intaking habit on the TC microbiota

**Figure 4:**
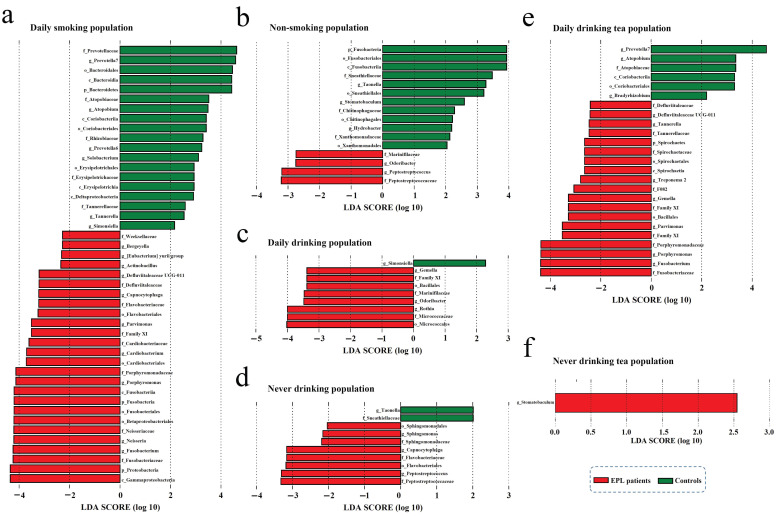
** The stratified analysis revealed microbial biomarkers of TC in cases and controls in certain populations with certain oral intake habit.** There were six types of oral intaking habits including daily smoking (**a**), non-smoking (**b**), daily drinking (**c**), never drinking (**d**), daily drinking tea (**e**), and never drinking tea (**f**).

To explore the effect of oral intaking habit on the TC microbiota and EPL risk, three credible self-reported habits (smoking, drinking, and drinking tea) were analyzed using the linear discriminant analysis effect size (LEfSe) to screen for potential TC microbiota **(Figure 4)**. In daily smokers, 26 TC bacterial taxa (including Gammaproteobacteria, Proteobacteria, and Fusobacteriaceae, etc.) were enriched in the cases and 19 TC bacterial taxa (including Prevotellaceae, *Prevotella 7*, and Bacteroidales, etc.) were enriched in the controls **(Figure 4a)**. In nonsmokers, four TC bacterial taxa (namely Peptostreptococcaceae,* Peptostreptococcus*,* Odoribacter*, and Marinifilaceae) were enriched in the cases, and 12 TC bacterial taxa (including Fusobacteria, Fusobacteriales, and Fusobacteriia, etc.) were enriched in the controls **(Figure 4b)**. For the daily drinkers, eight TC bacterial taxa (including Micrococcales, Micrococcaceae, and *Rothia*, etc.) were enriched in the cases and one TC bacterial taxon (*Simonsiella*) was enriched in the controls **(Figure 4c)**. For the nondrinkers, eight TC bacterial taxa (including Peptostreptococcaceae, *Peptostreptococcus*, and Flavobacteriales, etc.) were enriched in the cases and two TC bacterial taxa (namely *Taonella* and Sneathiellaceae) were enriched in the controls **(Figure 4d)**. For the daily tea drinkers, 19 TC bacterial taxa (including Fusobacteriaceae,* Fusobacterium*, and *Porphyromonas*, etc.) were enriched in the cases and six TC bacterial taxa (including *Prevotella 7*, *Atopobium*, and Atopobiaceae, etc.) were enriched in the controls **(Figure 4e)**. For the never tea drinkers, only *Stomatobaculum* was enriched in cases **(Figure 4f)**. These data suggest that the EPL-related microbiota is strongly affected by oral intaking habits.

In contrast, LEfSe analysis was also used to screen the potential TC microbiota related to specific oral intaking habits (such as daily drinkers and nondrinkers, daily smokers and nonsmokers, daily tea drinkers, and never tea drinkers). The results showed that the marker TC microbiota related to certain oral intaking habit was completely different between the cases and the controls **(Figure S2)**. For example, in the controls, four bacterial taxa in TC (namely *Granulicatella*, Carnobacteriaceae,* Catonella*, and Mollicutes RF39) were enriched in daily drinkers, and seven bacterial taxa in TC (including Moraxellaceae,* Selenomonas*, and Beijerinckiaceae, etc.) were enriched in people who never drank alcohol. However, in the cases, 17 bacterial taxa in TC (including Actinobacteria*,* Micrococcaceae, and *Rothia*, etc.) were enriched in people who drank every day, and 10 bacterial taxa in TC (including Prevotellaceae*,* Bacteroidia, and Bacteroidetes, etc.) were enriched in the never-drinking people.

The above results indicated that the TC flora was deeply affected by both EPL and oral intaking habits, which is consistent with previous results on gut microbiota 21.

### Relationship between oral intaking habit and TC microbiota

**Figure 5:**
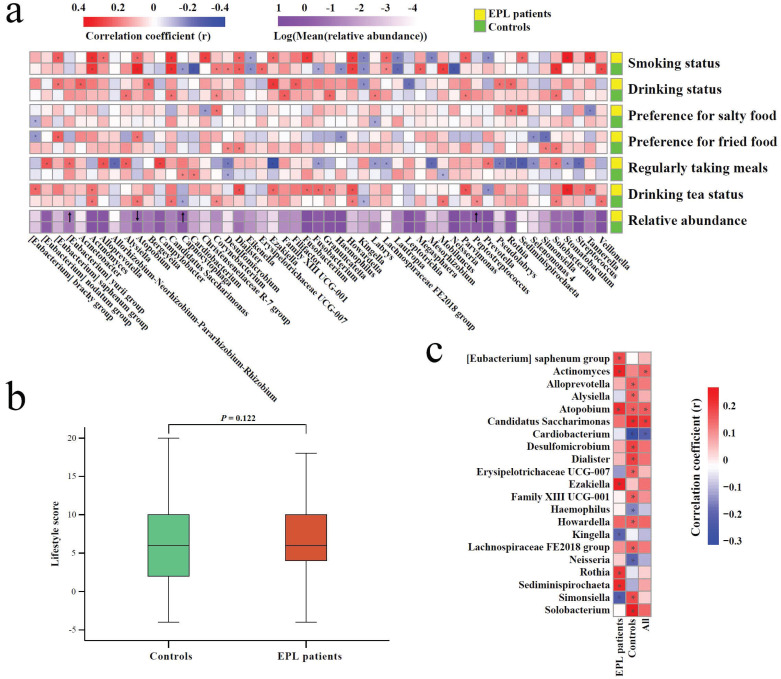
** Association among TC microbiota, oral intaking habit, and EPL.** (**a**) Correlation between TC microbiota and oral intaking habit degree in the cases and controls, respectively. (**b**) Different analyses of oral intaking habit scores between the cases and controls. (**c**) The correlation between oral intaking habit scores and TC bacterial genera in the cases, controls, and total population. In the heatmap, the red block indicates a positive correlation, the blue block indicates a negative correlation, and the asterisks in the block indicate *P* < 0.05.

In view of the complex effect of oral intaking habit on the TC microbiota and EPL risk, stratified analysis was carried out to comprehensively unfold the association among EPL risk, oral intaking habit, and TC microbiota **(Figure 5)**.

First, Spearman correlation analysis was conducted between the relative abundances of TC genera and the degree of six oral intaking habits (smoking, drinking, eating salty food, eating fried food, eating on time, and drinking tea) in the cases and the controls, respectively **(Figure 5a, Supplementary data for Figure 5a)**. The results showed that six genera (namely *Actinomyces*, *Atopobium*,* Candidatus Saccharimonas*,* Dialister*, *Howardella*, and* Lachnospiraceae FE2018 group*) were significantly positively correlated with smoking (*P* < 0.05), while three genera (namely *Eikenella*,* Kingella*, and* Lautropia*) were significantly negatively correlated with smoking (*P* < 0.05). *Simonsiella* was negatively correlated with eating fried food in cases (*P* < 0.05), but positively correlated with eating fried food in controls (*P* < 0.05); *Desulfomicrobium* was significantly negatively correlated with eating on time (*P* < 0.05); *Actinomyces* and* Howardella* were positively correlated with drinking tea (*P* < 0.05).

Second, everyone was given an oral intaking habit score according to the criteria (never = 0, seldom = 2, often = 4, everyday = 6), and cancer-risk factors (smoking, drinking, eating fried food, eating salty food, and eating on time) were calculated as positive scores, but the cancer-protective factor (drinking tea) was calculated as a negative score. The results of the Mann-Whitney *U* test showed that there was no significant difference between the cases and controls (*P* > 0.05) **(Figure 5b)**, which was consistent with the results of the oral intaking habit status of the population **(Table 1)**.

Finally, Spearman correlation analysis was used to screen for TC bacteria related to oral intaking habit scores. The results showed that *Atopobium* and *Actinomyces* were significantly positively correlated with oral intaking habit scores across the whole population, in the cases, and the controls (*P* < 0.05). In the controls and the whole population, *Candidatus Saccharimonas* was significantly positively correlated with oral intaking habit score (*P* < 0.05), while *Cardiobacterium* was significantly negatively correlated with oral intaking habit score (*P* < 0.05). *Simonsiella* was negatively correlated with oral intaking habit scores in the cases (*P* < 0.05), but positively correlated with oral intaking habit scores in the controls (*P* < 0.05) **(Figure 5c, Supplementary data for Figure 5c)**.

### Different analysis of predictive functions of TC microbiota

Based on the Kyoto Encyclopedia of Genes and Genomes (KEGG) predictive function of TC microflora, the Mann-Whitney *U* test analysis showed that thiamine metabolism and glycosphingolipid biosynthesis-lacto and neolacto series in EPL patients were higher than those in controls (*P* < 0.05), whereas butirosin and neomycin biosynthesis and streptomycin biosynthesis were significantly lower than those in the controls (*P* < 0.05) **(Table 3)**.

**Figure 6:**
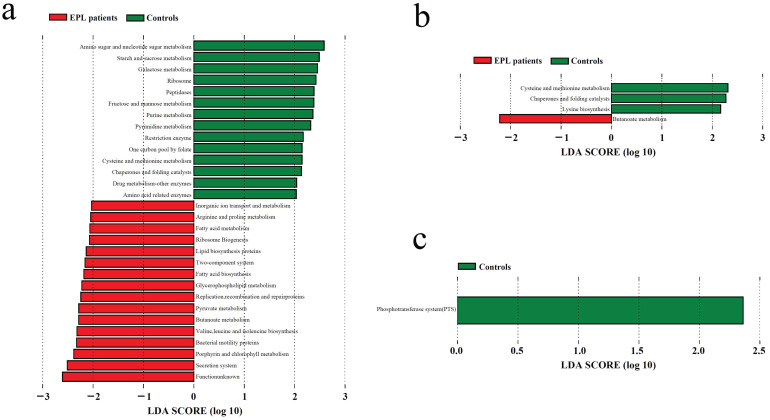
** LDA of the predictive functions based on TC microbiotain in the population with certain oral intaking habits.** (**a**) Daily smoking. (**b**) Daily drinking tea. (**c**) Never drinking tea.

LDA was used to determine the potential function of TC microbiota on three oral intaking habits (smoking, drinking, and drinking tea) in the cases and controls, respectively **(Figure 6)**. For daily smokers, 14 predictive functions (including amino sugar and nucleotide sugar metabolism, starch and sucrose metabolism, galactose metabolism) were enriched in controls, and 16 predictive functions (including secretion system, porphyrin and chlorophyll metabolism, and bacterial motility proteins) were enriched **(Figure 6a)**. For daily tea drinkers, three predictive functions (cysteine and methionine metabolism, chaperones and folding catalysts, and lysine biosynthesis) were enriched in controls, and one predictive function (butanoate metabolism) was enriched in cases **(Figure 6b)**. For people who never drank tea, a predictive functional taxon (phosphotransferase system) was enriched in the controls **(Figure 6c)**.

LDA was used to explore the effect of Hp infection on the predictive function of TC microbiota. The results suggest that chaperones and folding catalysts may be related to Hp infection in the controls, while phenylalanine, tyrosine, and tryptophan biosynthesis were related to non-Hp infection in cases **(Figure S3)**.

In addition, we also found that in cases and controls, distinctly different oral intaking habits (such as daily smoking and nonsmoking, daily drinking and nondrinking, daily tea drinking and never tea-drinking) exert deeply effects on the predictive function of TC microbiota **(Figure S4)**. For example, the secretion system, bacterial motility proteins, and other predictive functions were enriched in nonsmokers in controls **(Figure S4a)**, while bile secretion was significantly increased in daily drinkers in cases **(Figure S4f)**.

### Verification of the predictive function of TC microbiota

**Figure 7:**
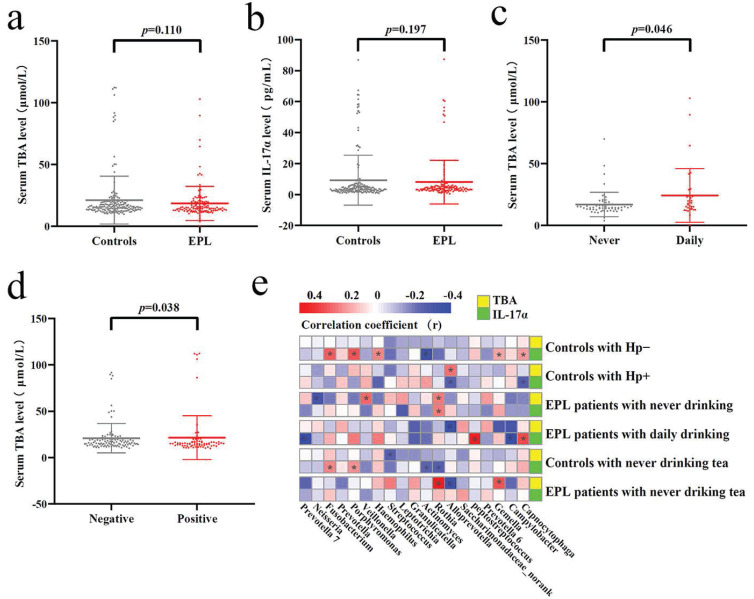
** Verification of the TC microbiota predictive functions.** Different analysis of serum TBA (**a**) and IL-17α (**b**) between the controls and cases. Significant difference of serum TBA between never and daily drinkers in cases (**c**). Significant difference of serum TBA between HP-negative and HP-positive controls (**d**). Correlation analysis was conducted between the dominant TC bacteria and serum TBA and IL-17α, the red square means positive correlation while the blue square means negative correlation, and the asterisk indicates significant correlation (*P* < 0.05) (**e**).

Tatsugami *et al.* 22 examined a significant positive correlation between bile acid concentration and the grades of atrophy/intestinal metaplasia in patients infected with Hp patients, and patients with a higher concentration of bile acid developed gastric cancer more frequently than those with a lower concentration. Karstens *et al.* 23 found that patients with EAC had lower serum IL-17α levels than healthy controls, and higher IL-17α levels might indicate a limited prognosis in patients with EAC. However, there was no significant difference in serum TBA and IL-17α levels between EPL patients and controls (*P* > 0.05) **(Figures 7a and 7b)**. Stratified analysis showed that the serum TBA in daily drinkers was significantly higher than that in nondrinkers in cases (*P* < 0.05) **(Figure 7c)**, while serum TBA in non-Hp-infected individuals was significantly higher than that in Hp-infected individuals in the controls (*P* < 0.05) **(Figure 7d)**. Spearman correlation analysis showed that TC *Alloprevotella* was positively correlated with serum TBA concentration (*P* < 0.05), but negatively correlated with IL-17α concentration (*P* < 0.05). TC *Rothia* was positively correlated with both serum TBA and IL-17α levels (*P* < 0.05) **(Figure 7e, Supplementary data for Figure 7e)**.

## Discussion

The esophagus is a muscular duct approximately 20-27 cm long, connects the mouth and stomach, and is normally wet with saliva. Its acid-base status is maintained at pH 7.0, but gastroesophageal reflux can distinctly drop the local pH from 7.0 to 2.0. Generally, there are few sedentary microorganisms in the esophageal mucosa, and the advancement of esophageal microbiota is greatly limited because of the invasive collection of the esophageal mucosa 24. Many factors, including gastric content reflux, oral microbiota, and lifestyle, deeply affect the esophageal mucosal microenvironment, especially the microbiota. With the development of culture-independent high-throughput sequencing technology, an increasing amount of evidences has shown that esophageal mucosal microbiota is associated with esophageal lesions, providing a new perspective for exploring the etiological mechanism of EC 25. The normal esophageal mucosal microbiota mainly consists of *Streptococcus*, *Granulicatella*,* Prevotella*, *Haemophilus*,* Staphylococcus*,* Veillonella*, *Propionibacterium*, and* Rothia*. In the case of Barrett's esophagus, the abundances of *Streptococcus*,* Granulicatella*, and* Propionibacterium* decreased in the esophageal mucosa, while the abundances of *Prevotella*,* Actinobacillus*,* Veillonella*, and* Leptotrichia* increased. When EAC developed, the abundance of *Streptococcus* and* Granulicatella* decreased in the esophageal mucosa, while the abundances of *Prevotella, Veillonella,* and* Leptotrichia* increased 26. Shao *et al*. 27 found that the microbial environments of ESCC and gastric cardia adenocarcinoma tissues were mainly composed of Firmicutes, Bacteroidetes, and Proteobacteria. Compared with non-tumor tissues, ESCC tumor tissues contained more Fusobacteria and less *Streptococcus,* and the relative abundance of *Fusobacterium* in ESCC tissues was positively correlated with increasing tumor stage. Snider *et al*. 28 found that the relative abundance of *Sphingomonas* in BE esophageal mucosa increased compared with controls, and patients with high-grade dysplasia or EAC had increased Enterobacteriaceae and *Akkermansia muciniphila*. Li *et al*. 29 used principal coordinate analysis to find that there were significant differences between the esophageal microbiomes of patients with ESCC and healthy controls, and found that *Clostridiales*, *Pseudomonas*, and Selenomonadales were the key taxa contributing to the altered microbiome in patients with ESCC. Yamamura *et al*. 30 detected the species *Fusobacterium nucleatum* in EC tissues, and found that the species was associated with shorter survival, suggesting a potential role as a prognostic biomarker. These results suggest that the esophageal microbiota is closely related to the development of EC.

However, collecting the esophageal mucosae is invasive and difficult to be accepted by most patients. Oral microbiota flowed through the esophagus with saliva and partially settled in the esophageal mucosal epithelium. A mountain study showed that oral microbiota is associated with many digestive diseases 31. Interestingly, Norder *et al.* 32 found that the oral microbiota was similar to the esophageal mucosa microbiota of healthy individuals. Mager *et al.* also found a high degree of similarity between saliva and TC microbiota, and most of the high abundances of OTUs appearing in saliva were derived from TC 33, 34. Therefore, TC may be the birthplace and an important distribution center of the oral microflora 16. Kageyama* et al*. 35 found that salivary microbiota may be associated with a variety of digestive tract tumors (tongue/pharynx, esophagus, stomach, and large intestine). Peters *et al*. 36 found that *Neisseria* and *Streptococcus pneumoniae* are associated with EAC risk. Zhao *et al*. 37 found that *Neisseria*, *Prevotella*, and *Veillonella* may be potential new biomarkers of EC. Kawasaki *et al.* 1 found that dental plaque *Treponema denticola, Streptococcus anginosus*, and saliva *Aggregatibacter actinomycetemcomitans* were significantly associated with the risk of EC. These results strongly suggest that oral microbiota, including TC microbiota, may provide potential diagnostic markers for esophageal lesions.

In this study, EPL patients had significantly decreased diversity and richness of TC microbiota, but no significant differences were observed. Li *et al*. 29 found that the richness and diversity of esophageal microbiota decreased significantly in patients with EC, and Chen *et al.* 38 found that the diversity of microbiota in the saliva of patients with ESCC also decreased significantly. LDA showed that three TC genera (*[Eubacterium] yurii group*, *Peptostreptococcus*, and *Capnocytophaga*) increased in EPL patients. Lee *et al*. 39 found that the relative abundances of *Peptostreptococcus*,* Bacillus*, and* Parvimonas* in oral cancer mucosa were significantly higher than that in precancerous mucosa. Yan *et al*. 40 found that the relative abundances of *Capnocytophaga* and *Veillonella* in the saliva of patients with lung cancer increased significantly. These results suggest that TC* Peptostreptococcus* and *Capnocytophaga* might be potential marker microbiota related to EPL risk.

Latest epidemiological evidence has confirmed that cigarette smoke increases the overall risk of cancer, especially cancers in the lungs, bronchi, trachea, larynx, colon, pancreas, and cervix 41. A case-control study showed that education level, sleep quality, smoking, exposure to several foods and seasonings, preference for specific tastes, and various lifestyles were associated with gastric adenocarcinoma 42. The occurrence and development of EC involve various factors 43, such as aging, diet, and geographical environment, which also affect the human microbiome. Whether in the cases or the controls, the relative abundances of six genera (*Actinomyces*, *Atopobium*, *Candidatus Saccharimonas*, *Dialister*,* Howardella*, and *Lachnospiraceae FE2018 group*) were significantly positively correlated with smoking frequency, while the relative abundances of three genera (*Eikenella*, *Kingella*, and* Lautropia*) were significantly negatively correlated with smoking frequency. Zhang *et al*. 44 found that smoking significantly improved several gut bacterial genera (*Actinomyces*,* Collinsella*,* Lachnospiraceae_UCG-008*, and *Paraprevotella*) in patients with ankylosing spondylitis. Wu *et al*. 45 found that smoking significantly improved several oral bacterial genera, such as *Atopobium, Lactobacillus,* and *Streptococcus*. Shchipkova *et al*. 46 found that smoking significantly improved several subgingival bacteria, including *Treponema socranskii*,* Dialister pneumosintes*, and* Peptostreptococcus*. Vallès *et al.* 47 found that cigarette smokers lacked oral *Neisseria*, *Eikenella*, *Aggregatibacter*, *Actinobacillus*,* Haemophilus*, and* Lautropia*. The present study found that smoking decreased the relative abundances of TC *Neisseria*, *Capnocytophaga*, and *Lautropia* in controls, and a similar phenomenon was observed in salivary microbiota in the Jordanian 48, Hungarian 49, and American populations 50. Thus, it can be concluded that smoking could increase the abundance of *Actinomyces*, *Atopobium*, and* Dialister*, but decreased the abundance of *Eikenella* and *Lautropia.* Furthermore, these inconsistent results suggest that TC microbiota is deeply influenced by lifestyle and diseases, and the most important point is that standardizing the research environment and methods gives impetus to the repeatability of such research results.

Bacterial symbiosis can reflect the pathophysiological state, and the imbalance of the microbiota is an important characteristic of EC 51. Here, we found that the development of EPL weakened the TC commensal network of *Capnocytophaga*, *Fusobacterium*, *Porphyromonas*, *Haemophilus*, and* Atopobium*. Lu *et al*. 52 found that well-maintained subjects with a history of periodontitis had a more complex and robust symbiotic network with more pathogenic bacteria (such as *Porphyromonas*,* Prevotella*, *Tannerella*, and* Fusobacterium*) than the healthy controls. The results suggest that the EPL weakened the symbiotic networks and reduced the symbiotic complexity of the oral microbiota, in addition to the TC microbiota.

Several recent studies have shown that the gut microbiota participates in the biosynthetic pathway of bile acids 53, 54. This study found that daily drinkers had an enhanced predictive function for TC microbiota and bile secretion, which was verified by serum TBA levels. Early studies have found that alcohol can stimulate the rapid synthesis of bile acids 55. Wang *et al*. 56 observed that acidified bile acids could induce tumor progression and telomerase activity in gastric cancer both *in vivo* and *in vitro*. Tatsugami *et al*. 22 found that the bile acid of gastric juice contributed to the progression of histological atrophy and intestinal metaplasia without inflammatory cell infiltration, followed by carcinogenesis in patients infected with Hp. Donepudi *et al*. 57 found that chronic heavy drinking reduced the expression of cholesterol 7α-hydroxylase in the liver and increased the reabsorption of intestinal bile acid, while the lack of expression of cholesterol 7α-hydroxylase in the liver was the key factor in alcohol-induced liver injury. Hartmann *et al*. 58 found that mice with chronic ethanol intake showed an overrepresentation of bacterial genomic DNA encoding choloylglycine hydrolase, which could deconjugate bile acids and increase the level of free bile acids in the intestines, and such altered bile acids could distinctly reduce ethanol-induced liver disease. These results suggest that bile acid metabolism plays an important role in the occurrence of EPL linked to daily drinking and altered TC microbiota.

The limitations of this study were as follows: 1) oral intaking habit was mainly dependent on the reports of patients, which was subjective; 2) the results need to be further verified in more regional and larger prospective studies. In any case, this study explored the quantitative pattern of oral intaking habits changing the TC microbiota, and the results provide a new perspective on the relationship between oral intaking habit and TC microbiota in EPL patients.

## Supplementary Material

Supplementary figures and table.

## Figures and Tables

**Table 1:** Clinical characteristics of EPL patients and Controls

Characteristics		Controls (n=176)	EPL patients (n=123)	t/χ^2^	*P*
Age		58.99±7.18	59.71±6.43	0.889 ^a^	0.375
BMI		24.82±3.29	24.44±2.64	1.080^ a^	0.281
Gender	Male	96(54.5%)	71(57.7%)	0.297 ^b^	0.586
	Female	80(45.5%)	52(42.3%)		
Smoking status	Never	113(64.2%)	81(65.9%)	11.690 ^b^	0.009
	Seldom	7(4.0%)	1(0.8%)		
	Often	1(0.6%)	8(6.5%)		
	Daily	55(31.3%)	33(26.8%)		
Drinking status	Never	91(51.7%)	60(48.8%)	10.290 ^b^	0.016
	Seldom	45(25.6%)	18(14.6)		
	Often	14(8.0%)	11(8.9%)		
	Daily	26(14.8%)	34(27.6%)		
Preference for salty food	Never	27(15.3%)	13(10.6%)	3.490^ b^	0.322
	Seldom	109(61.9%)	72(58.5%)		
	Often	23(13.1%)	24(19.5%)		
	Daily	17(9.7%)	14(11.4%)		
Preference for fried food	Never	54(30.7%)	24(19.5%)	4.777^ b^	0.092
	Seldom	118(67.0%)	95(77.2%)		
	Often	4(2.3%)	4(3.3%)		
Regularly taking meals	Seldom	4(2.3%)	2(1.6%)	0.377^ b^	0.828
	Often	17(9.7%)	10(8.1%)		
	Daily	155(88.1%)	111(90.2%)		
Drinking tea status	Never	96(54.5%)	53(43.1%)	6.033^ b^	0.110
	Seldom	42(23.9%)	44(35.8%)		
	Often	12(6.8%)	6(4.9%)		
	Daily	26(14.8%)	20(16.3%)		
Hp-infection status	Positive	72(40.9%)	50(40.7%)	0.002^ b^	0.964
	Negative	104(59.1%)	73(59.3%)		

^a^ Refers to students' t test, ^b^ refers to Chi-square test.

**Table 2:** Alpha diversity analysis between EPL patients and controls [Median (P25, P75)]

	Ace	Chao	Shannon	Simpson	Observed OTUs
Controls (n=176)	1483(1242,1790)	1425(1185,1575)	3.967(3.793,4.175)	0.049(0.040,0.062)	982.5(821.5,1098)
EPL patients (n=123)	1478(1223,1729)	1366(1165,1569)	3.943(3.746,4.150)	0.050(0.041,0.065)	947.0(807.0,1078)
Z(*P*)	0.754(0.451)	0.705(0.481)	0.890(0.373)	0.454(0.650)	1.079(0.281)

**Table 3:** Different analysis of predictive functions between EPL patients and Controls

Functions	Controls (n=176)	EPL patients (n=123)	Z(*P*)
Butirosin and neomycin biosynthesis	0.0500±0.0089	0.0477±0.0074	2.102(0.036)
Streptomycin biosynthesis	0.3210±0.0207	0.3165±0.0210	2.089(0.037)
Glycosphingolipid biosynthesis-lacto and neolacto series	0.0001±0.0001	0.0001±0.0001	2.036(0.042)
Thiamine metabolism	0.4896±0.0249	0.4961±0.0215	2.482(0.013)

## Abbreviations

EC: esophageal cancer
TC: tongue coating
EPL: esophageal precancerous lesions
TBA: total bile acid
IL-17α: interleukin-17α
ELISA: enzyme-linked immunosorbent assay
Hp: *Helicobacter pylori*
ESCC: esophageal squamous cell carcinoma
EAC: esophageal adenocarcinoma
OTUs: operational taxonomic units
LDA: linear discriminant analysis
LEfSe: linear discriminant analysis effect size
KEGG: Kyoto Encyclopedia of Genes and Genomes
