# Supplementary material for: Influence of Oral Intaking Habit on Tongue Coating Microbiota in Patients with Esophageal Precancerous Lesions
Source: J Cancer. 2022 Jan 16;13(4):1168–80. doi: 10.7150/jca.67068 (PMC8899384; doi:10.7150/jca.67068)
Supplement: Supplementary file 1 — Supplementary figures and table. [file jcav13p1168s1.pdf]

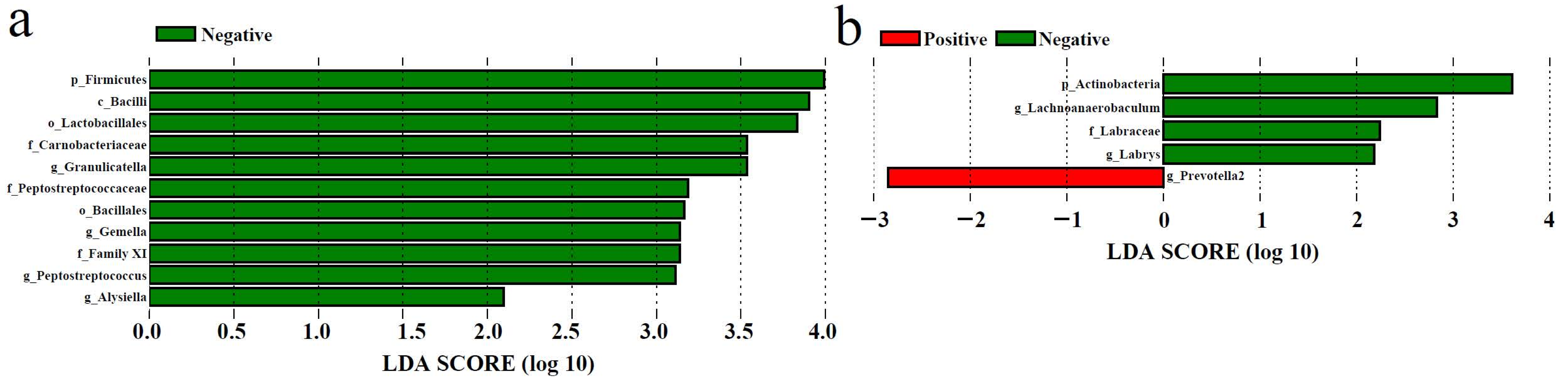

a
Negative
p_Firmicutes
c_Bacilli
o_Lactobacillales
f_Carnobacteriaceae
g_Granulicatella
f_Peptostreptococcaceae
o_Bacillales
g_Gemella
f_Family XI
g_Peptostreptococcus
g_Alysiella
0.0 0.5 1.0 1.5 2.0 2.5 3.0 3.5 4.0
LDA SCORE (log 10)
b
Positive
Negative
p_Actinobacteria
g_Lachnoanaerobaculum
f_Labraceae
g_Labrys
g_Prevotella2
−3 −2 −1 0 1 2 3 4
LDA SCORE (log 10)

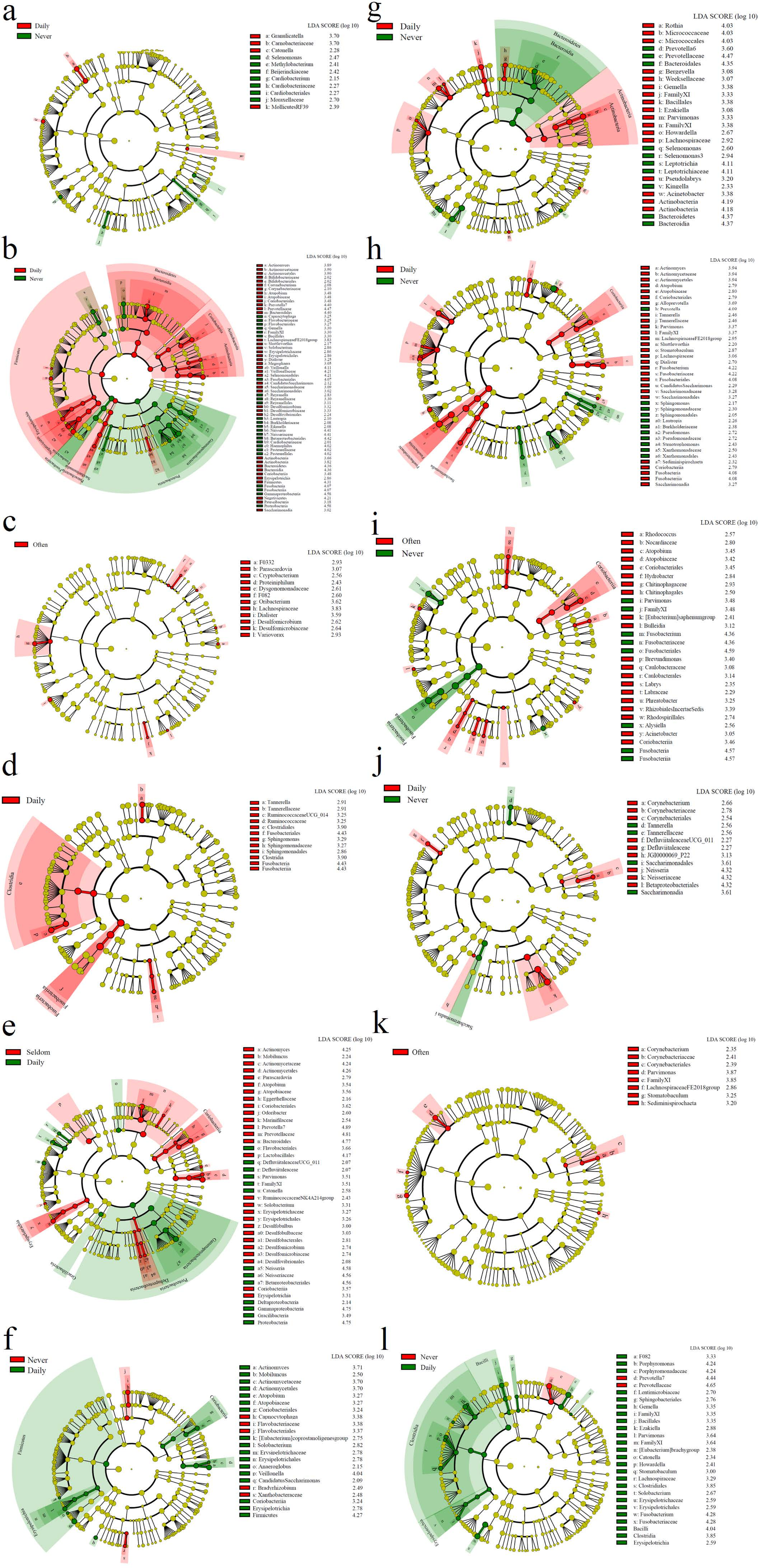

a
Daily
Never
LDA SCORE (log 10)
a: Granulicatella 3.70
b: Carnobacteriaceae 3.70
c: Catonella 2.28
d: Selenomonas 2.47
e: Methylobacterium 2.41
f: Beijerinckiaceae 2.42
g: Cardiobacterium 2.15
h: Cardiobacteriaceae 2.27
i: Cardiobacteriales 2.27
j: Moraxellaceae 2.70
k: MollicutesRF39 2.39
b
Daily
Never
LDA SCORE (log 10)
c
Often
LDA SCORE (log 10)
a: F0332 2.93
b: Parascardovia 3.07
c: Cryptobacterium 2.56
d: Proteiniphilum 2.43
e: Dysgonomonadaceae 2.61
f: F082 2.60
g: Oribacterium 3.62
h: Lachnospiraceae 3.83
i: Dialister 3.59
j: Desulfomicrobium 2.62
k: Desulfomicrobiaceae 2.64
l: Variovorax 2.93
d
Daily
LDA SCORE (log 10)
a: Tannerella 2.91
b: Tannerellaceae 2.91
c: RuminococcaceaeUCG_014 3.25
d: Ruminococcaceae 3.25
e: Clostridiales 3.90
f: Fusobacteriales 4.43
g: Sphingomonas 3.29
h: Sphingomonadaceae 3.27
i: Sphingomonadales 2.86
Clostridia 3.90
Fusobacteria 4.43
Fusobacteriia 4.43
Clostridia
Fusobacteriia
e
Seldom
Daily
LDA SCORE (log 10)
a: Actinomyces 4.25
b: Mobiluncus 2.24
c: Actinomycetaceae 4.24
d: Actinomycetales 4.26
e: Parascardovia 2.79
f: Atopobium 3.54
g: Atopobiaceae 3.56
h: Eggerthellaceae 2.16
i: Coriobacteriales 3.62
j: Odoribacter 2.60
k: Marinifilaceae 2.54
l: Prevotella7 4.89
m: Prevotellaceae 4.81
n: Bacteroidales 4.77
o: Flavobacteriales 3.66
p: Lactobacillales 4.17
q: DefluviitaleaceaeUCG_011 2.07
r: Defluviitaleaceae 2.07
s: Parvimonas 3.51
t: FamilyXI 3.51
u: Catonella 2.58
v: RuminococcaceaeNK4A214group 2.43
w: Solobacterium 3.31
x: Erysipelotrichaceae 3.27
y: Erysipelotrichales 3.26
z: Desulfobulbus 3.00
a0: Desulfobulbaceae 3.03
a1: Desulfobacterales 2.81
a2: Desulfomicrobium 2.74
a3: Desulfomicrobiaceae 2.74
a4: Desulfovibrionales 2.08
a5: Neisseria 4.58
a6: Neisseriaceae 4.56
a7: Betaproteobacteriales 4.56
Coriobacteriia 3.57
Erysipelotrichia 3.31
Deltaproteobacteria 2.14
Gammaproteobacteria 4.75
Gracilibacteria 3.49
Proteobacteria 4.75
f
Never
Daily
LDA SCORE (log 10)
a: Actinomyces 3.71
b: Mobiluncus 2.50
c: Actinomycetaceae 3.70
d: Actinomycetales 3.70
e: Atopobium 3.27
f: Atopobiaceae 3.27
g: Coriobacteriales 3.24
h: Capnocytophaga 3.38
i: Flavobacteriaceae 3.38
j: Flavobacteriales 3.37
k: [Eubacterium]coprostanoligenesgroup 2.75
l: Solobacterium 2.82
m: Erysipelotrichaceae 2.78
n: Erysipelotrichales 2.78
o: Anaeroglobus 2.15
p: Veillonella 4.04
q: CandidatusSaccharimonas 2.09
r: Bradyrhizobium 2.49
s: Xanthobacteraceae 2.48
Coriobacteriia 3.24
Erysipelotrichia 2.78
Firmicutes 4.27
g
Daily
Never
LDA SCORE (log 10)
a: Rothia 4.03
b: Micrococcaceae 4.03
c: Micrococcales 4.03
d: Prevotella6 3.60
e: Prevotellaceae 4.47
f: Bacteroidales 4.35
g: Bergeyella 3.08
h: Weeksellaceae 3.07
i: Gemella 3.38
j: FamilyXI 3.33
k: Bacillales 3.38
l: Ezakiella 3.08
m: Parvimonas 3.33
n: FamilyXI 3.38
o: Howardella 2.67
p: Lachnospiraceae 2.92
q: Selenomonas 2.60
r: Selenomonas3 2.94
s: Leptotrichia 4.11
t: Leptotrichiaceae 4.11
u: Pseudolabrys 3.20
v: Kingella 2.33
w: Acinetobacter 3.38
Actinobacteria 4.19
Actinobacteria 4.18
Bacteroidetes 4.37
Bacteroidia 4.37
h
Daily
Never
LDA SCORE (log 10)
i
Often
Never
LDA SCORE (log 10)
a: Rhodococcus 2.57
b: Nocardiaceae 2.80
c: Atopobium 3.45
d: Atopobiaceae 3.42
e: Coriobacteriales 3.45
f: Hydrobacter 2.84
g: Chitinophagaceae 2.93
h: Chitinophagales 2.50
i: Parvimonas 3.48
j: FamilyXI 3.48
k: [Eubacterium]saphenumgroup 2.41
l: Bulleidia 3.12
m: Fusobacterium 4.36
n: Fusobacteriaceae 4.36
o: Fusobacteriales 4.59
p: Brevundimonas 3.40
q: Caulobacteraceae 3.08
r: Caulobacterales 3.14
s: Labrys 2.35
t: Labraceae 2.29
u: Phreatobacter 3.25
v: RhizobialesIncertaeSedis 3.39
w: Rhodospirillales 2.74
x: Alysiella 2.56
y: Acinetobacter 3.05
Coriobacteriia 3.46
Fusobacteria 4.57
Fusobacteriia 4.57
j
Daily
Never
LDA SCORE (log 10)
a: Corynebacterium 2.66
b: Corynebacteriaceae 2.78
c: Corynebacteriales 2.54
d: Tannerella 2.56
e: Tannerellaceae 2.56
f: DefluviitaleaceaeUCG_011 2.27
g: Defluviitaleaceae 2.27
h: JGI0000069_P22 3.13
i: Saccharimonadales 3.61
j: Neisseria 4.32
k: Neisseriaceae 4.32
l: Betaproteobacteriales 4.32
Saccharimonadia 3.61
k
Often
LDA SCORE (log 10)
a: Corynebacterium 2.35
b: Corynebacteriaceae 2.41
c: Corynebacteriales 2.39
d: Parvimonas 3.87
e: FamilyXI 3.85
f: LachnospiraceaeFE2018group 2.86
g: Stomatobaculum 3.25
h: Sediminispirochaeta 3.20
l
Never
Daily
LDA SCORE (log 10)

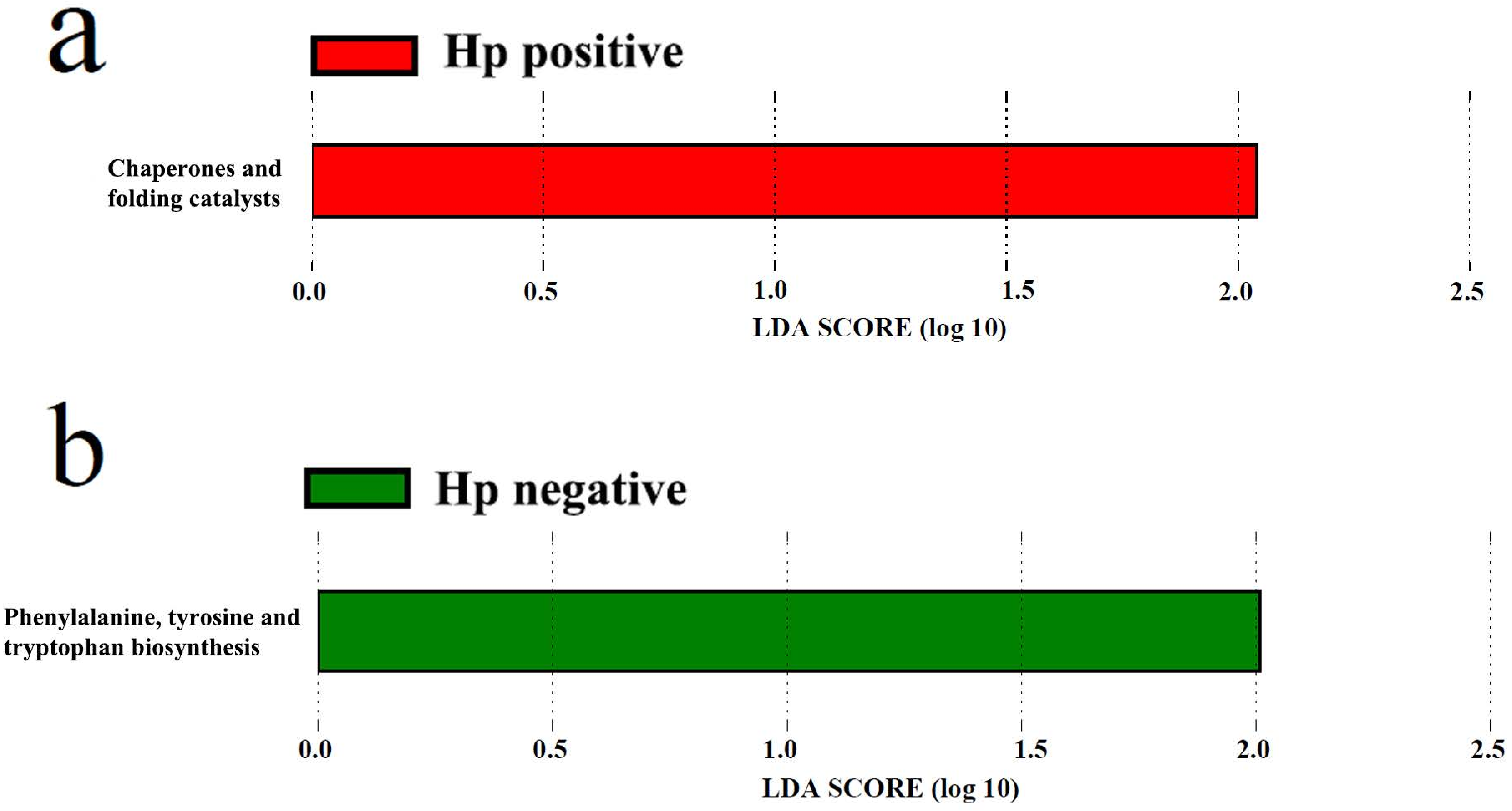
a
Hp positive
Chaperones and folding catalysts
0.0
0.5
1.0
1.5
2.0
2.5
LDA SCORE (log 10)
b
Hp negative
Phenylalanine, tyrosine and tryptophan biosynthesis
0.0
0.5
1.0
1.5
2.0
2.5
LDA SCORE (log 10)

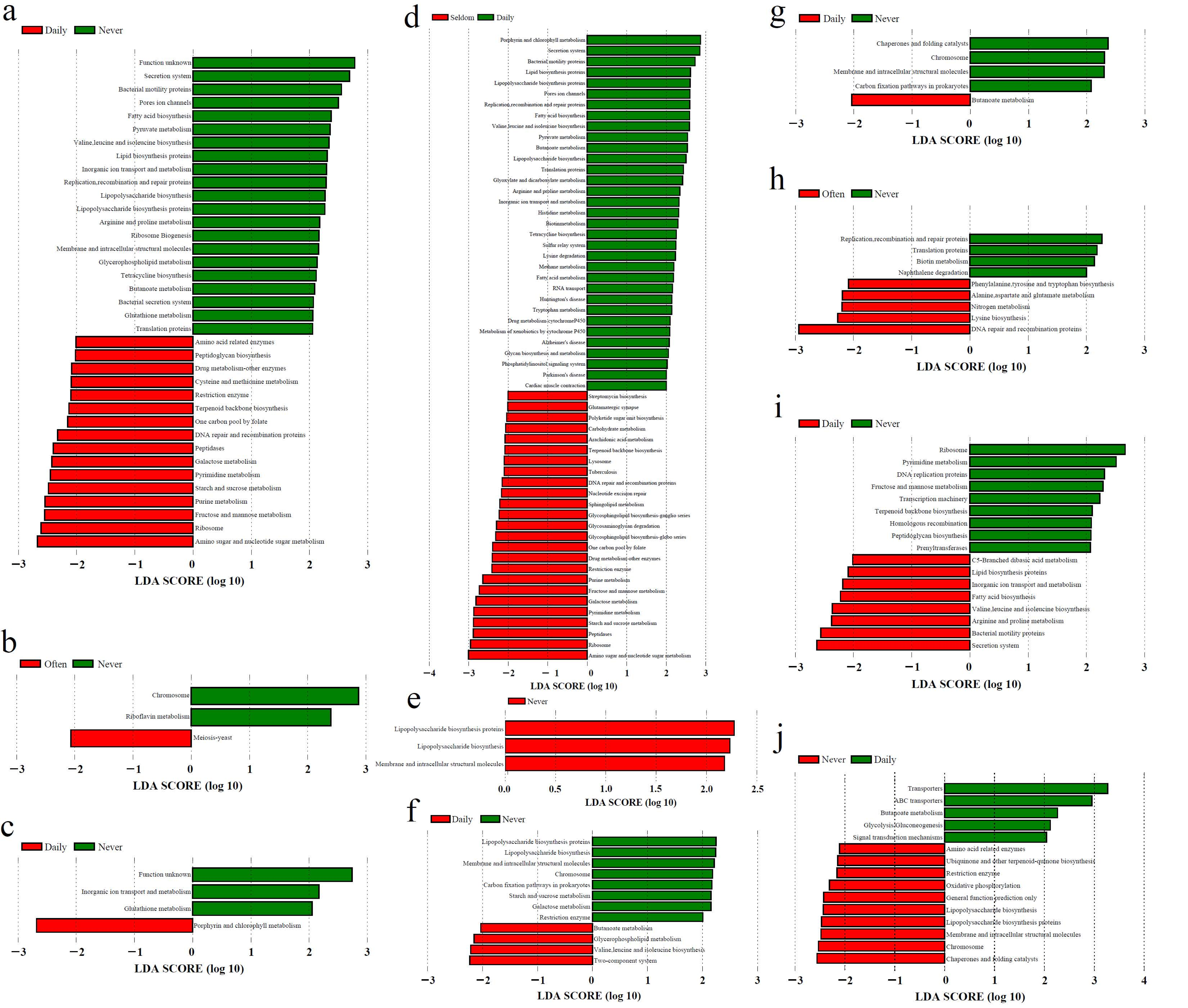
a
Daily
Never
Function unknown
Secretion system
Bacterial motility proteins
Pores ion channels
Fatty acid biosynthesis
Pyruvate metabolism
Valine,leucine and isoleucine biosynthesis
Lipid biosynthesis proteins
Inorganic ion transport and metabolism
Replication,recombination and repair proteins
Lipopolysaccharide biosynthesis
Lipopolysaccharide biosynthesis proteins
Arginine and proline metabolism
Ribosome Biogenesis
Membrane and intracellular structural molecules
Glycerophospholipid metabolism
Tetracycline biosynthesis
Butanoate metabolism
Bacterial secretion system
Glutathione metabolism
Translation proteins
Amino acid related enzymes
Peptidoglycan biosynthesis
Drug metabolism-other enzymes
Cysteine and methionine metabolism
Restriction enzyme
Terpenoid backbone biosynthesis
One carbon pool by folate
DNA repair and recombination proteins
Peptidases
Galactose metabolism
Pyrimidine metabolism
Starch and sucrose metabolism
Purine metabolism
Fructose and mannose metabolism
Ribosome
Amino sugar and nucleotide sugar metabolism
−3 −2 −1 0 1 2 3
LDA SCORE (log 10)
b
Often
Never
Chromosome
Riboflavin metabolism
Meiosis-yeast
−3 −2 −1 0 1 2 3
LDA SCORE (log 10)
c
Daily
Never
Function unknown
Inorganic ion transport and metabolism
Glutathione metabolism
Porphyrin and chlorophyll metabolism
−3 −2 −1 0 1 2 3
LDA SCORE (log 10)
d
Seldom
Daily
Porphyrin and chlorophyll metabolism
Secretion system
Bacterial motility proteins
Lipid biosynthesis proteins
Lipopolysaccharide biosynthesis proteins
Pores ion channels
Replication,recombination and repair proteins
Fatty acid biosynthesis
Valine,leucine and isoleucine biosynthesis
Pyruvate metabolism
Butanoate metabolism
Lipopolysaccharide biosynthesis
Translation proteins
Glyoxylate and dicarboxylate metabolism
Arginine and proline metabolism
Inorganic ion transport and metabolism
Histidine metabolism
Biotinmetabolism
Tetracycline biosynthesis
Sulfur relay system
Lysine degradation
Methane metabolism
Fatty acid metabolism
RNA transport
Huntington's disease
Tryptophan metabolism
Drug metabolism-cytochromeP450
Metabolism of xenobiotics by cytochrome P450
Alzheimer's disease
Glycan biosynthesis and metabolism
Phosphatidylinositol signaling system
Parkinson's disease
Cardiac muscle contraction
Streptomycin biosynthesis
Glutamatergic synapse
Polyketide sugar unit biosynthesis
Carbohydrate metabolism
Arachidonic acid metabolism
Terpenoid backbone biosynthesis
Lysosome
Tuberculosis
DNA repair and recombination proteins
Nucleotide excision repair
Sphingolipid metabolism
Glycosphingolipid biosynthesis-ganglio series
Glycosaminoglycan degradation
Glycosphingolipid biosynthesis-globo series
One carbon pool by folate
Drug metabolism-other enzymes
Restriction enzyme
Purine metabolism
Fructose and mannose metabolism
Galactose metabolism
Pyrimidine metabolism
Starch and sucrose metabolism
Peptidases
Ribosome
Amino sugar and nucleotide sugar metabolism
−4 −3 −2 − 1 0 1 2 3
LDA SCORE (log 10)
e
Never
Lipopolysaccharide biosynthesis proteins
Lipopolysaccharide biosynthesis
Membrane and intracellular structural molecules
0.0 0.5 1.0 1.5 2.0 2.5
LDA SCORE (log 10)
f
Daily
Never
Lipopolysaccharide biosynthesis proteins
Lipopolysaccharide biosynthesis
Membrane and intracellular structural molecules
Chromosome
Carbon fixation pathways in prokaryotes
Starch and sucrose metabolism
Galactose metabolism
Restriction enzyme
Butanoate metabolism
Glycerophospholipid metabolism
Valine,leucine and isoleucine biosynthesis
Two-component system
−3 −2 −1 0 1 2 3
LDA SCORE (log 10)
g
Daily
Never
Chaperones and folding catalysts
Chromosome
Membrane and intracellular structural molecules
Carbon fixation pathways in prokaryotes
Butanoate metabolism
−3 −2 −1 0 1 2 3
LDA SCORE (log 10)
h
Often
Never
Replication,recombination and repair proteins
Translation proteins
Biotin metabolism
Naphthalene degradation
Phenylalanine,tyrosine and tryptophan biosynthesis
Alanine,aspartate and glutamate metabolism
Nitrogen metabolism
Lysine biosynthesis
DNA repair and recombination proteins
−3 −2 −1 0 1 2 3
LDA SCORE (log 10)
i
Daily
Never
Ribosome
Pyrimidine metabolism
DNA replication proteins
Fructose and mannose metabolism
Transcription machinery
Terpenoid backbone biosynthesis
Homologous recombination
Peptidoglycan biosynthesis
Prenyltransferases
C5-Branched dibasic acid metabolism
Lipid biosynthesis proteins
Inorganic ion transport and metabolism
Fatty acid biosynthesis
Valine,leucine and isoleucine biosynthesis
Arginine and proline metabolism
Bacterial motility proteins
Secretion system
−3 −2 −1 0 1 2 3
LDA SCORE (log 10)
j
Never
Daily
Transporters
ABC transporters
Butanoate metabolism
Glycolysis Gluconeogenesis
Signal transduction mechanisms
Amino acid related enzymes
Ubiquinone and other terpenoid-quinone biosynthesis
Restriction enzyme
Oxidative phosphorylation
General function prediction only
Lipopolysaccharide biosynthesis
Lipopolysaccharide biosynthesis proteins
Membrane and intracellular structural molecules
Chromosome
Chaperones and folding catalysts
−3 −2 −1 0 1 2 3 4
LDA SCORE (log 10)

Table S1. Comparative analysis of the genus level of tongue coating flora between EPL patients and Controls [Relative abundance (%), Median (P25, P75)]

Bacteria	Controls	EPL patients	Z(*P*)
[Eubacterium] yurii group	0.05042(0.00630,0.13551)	0.09454(0.01891,0.18278)	2.653(0.008)
Atopobium	0.22375(0.08824,0.57828)	0.17018(0.07563,0.40338)	2.022(0.043)
Capnocytophaga	0.41283(0.13236,1.07462)	0.62398(0.23951,1.57570)	2.318(0.020)
Comamonas	0.00630(0.00000,0.01261)	0.00630(0.00000,0.01891)	2.176(0.030)
Defluviitaleaceae UCG-011	0.00630(0.00000,0.03151)	0.01261(0.00000,0.03782)	2.252(0.024)
Hydrobacter	0.00000(0.00000,0.00630)	0.00000(0.00000,0.00000)	2.076(0.038)
Odoribacter	0.00000(0.00000,0.00000)	0.00000(0.00000,0.00000)	2.312(0.021)
Peptostreptococcus	1.02735(0.53574,1.60563)	1.17862(0.73112,1.84672)	1.989(0.047)
Taonella	0.00000(0.00000,0.00000)	0.00000(0.00000,0.00000)	2.065(0.039)
